# Interactive Low Back Pain Intervention Module Based on the Back School Program: A Cluster-Randomized Experimental Study Evaluating Its Effectiveness among Nurses in Public Hospitals

**DOI:** 10.3390/ijerph17165916

**Published:** 2020-08-14

**Authors:** Mohd Ismail Ibrahim, Izani Uzair Zubair, Mohd Nazri Shafei, Mohd Izmi Ahmad, Najib Majdi Yaacob

**Affiliations:** 1 Department of Community Medicine, School of Medical Sciences, Health Campus, Universiti Sains Malaysia, Kubang Kerian, Kelantan 16150, Malaysia; ismaildr@usm.my (M.I.I.); drnazri@usm.my (M.N.S.); 2 Penang Health State Department, 33 Pengkalan Weld, George Town, Penang 10300, Malaysia; izani_um@yahoo.com; 3 Hospital Pulau Pinang, Jalan Residensi, George Town, Penang 10990, Malaysia; mohdizmi@yahoo.com.au; 4 Units of Biostatistics and Research Methodology, School of Medical Sciences, Health Campus, Universiti Sains Malaysia, Kubang Kerian, Kelantan 16150, Malaysia

**Keywords:** low back pain, nurses, back school program, Oswestry Disability Questionnaire

## Abstract

The prevalence of low back pain (LBP) among nurses is high. The main aim of this study was to evaluate the effectiveness of an interactive LBP module based on the Back School Program in improving Oswestry Disability Scores (ODSs) among nurses in government hospitals in Penang, Malaysia. A cluster-randomized experimental study was conducted within four public hospitals. These hospitals were randomized to intervention and control groups. A total of 284 nurses from the selected hospitals were randomly selected (142 in each group). An interactive LBP intervention module based on the Back School Program was designed and prescribed. Both the intervention and control groups were assessed using the Oswestry Disability Questionnaire at baseline and at the end of the third and sixth weeks. Out of 284 participants, 281 completed this study. A between-group comparison revealed that ODSs were significantly lower in the intervention group than in the control group at the ends of the third (*p* = 0.006) and sixth weeks (*p* < 0.001). Within-group changes revealed a significant reduction in ODSs within the intervention group from baseline to the third (*p* < 0.001) and sixth weeks (*p* < 0.001) of the intervention. This simple interactive LBP module was effective in reducing symptoms of LBP among nurses as early as three weeks, and this effect was sustained until the sixth week of the intervention.

## 1. Introduction

Low back pain (LBP) is a common symptom with the potential to cause severe disabilities that constitute a major public health problem worldwide. According to the Global Burden of Disease 2010 Study, LBP ranked sixth from the perspective of the disability-adjusted life year. Globally, nearly 9.4% of people have experienced LBP; further, LBP is the main cause of disability and nonattendance from work in as many as 187 countries [1].

Physical and mental demands may cause nurses to leave their jobs. Nurses may be exposed to several hazards that may result in LBP while providing patient care. An overview of five nations revealed that 17% to 39% of nurses wanted to quit their employment because of the physical and mental commitments required by their jobs [2]. The prevalence of back illness has been reported to be six times higher among nurses than among other health professionals. This, in turn, increases work nonattendance and job-related disability costs [3,4]. Another consequence of LBP among nursing staff is disruption to the quality of patient care. In studies by Daraiseh et al. [5] and Byrns et al. [6], the effect of LBP on nurse turnover was found to cause shortages of nurses that jeopardize or weaken the quality of nursing care, which is directly related to adverse patient outcomes. A study conducted by Wong et al. [7] in Sarawak, a state of east Malaysia, showed that the prevalence of LBP was 38.8% among staff nurses, 19.0% among community nurses, and 13.7% among doctors. Wong, Teo, and Kyaw [7] further elucidated that LBP can occur for several reasons, including improper body posture and carrying of objects and patients. Sociodemographic, working environment, psychological, and lifestyle factors may differ between working environments. Therefore, the outcomes of any given treatment approach may vary slightly between localities.

Today, nursing associations are working toward the advancement of wellbeing and disease anticipation among nurses. The International Council of Nurses (ICN) has emphasized the importance of establishing work environments that are conducive to positive patient outcomes. The ICN has also recommended the establishment of national and local organizational policies focused on injury prevention, including prevention of LBP among nurses [8]. Strategies to overcome LBP are plentiful and include coping techniques and intervention programs such as physiotherapy, active treatments (e.g., Back School, core-strengthening exercises, physical fitness programs, and the McKenzie method), and passive treatment modalities (e.g., manual therapy, soft tissue techniques, traction, electrotherapy, and heat and cold therapies). Intervention programs for the treatment of LBP have been evaluated in terms of their effectiveness in reducing occurrences of back injuries among nurses [9]. However, very few outcomes have been reported for these intervention programs [10,11]. Nurses’ knowledge concerning back injuries and prevention strategies is an important factor in the implementation of viable intervention programs. 

The Back School Program is a method of preventing recurrent episodes of LBP developed by Mariane Zachrisson Forssel in 1980 [12]. This intervention module was originally developed as an education and training program and involves exercise and group lessons monitored by a physiotherapist or physician [13]. Common topics of the educational component of the Back School Program include the anatomy of the spine, theories of the etiology of LBP, the function of backbone, ergonomics, exercise, complications of back pain, and changing unpleasant beliefs about back pain. This model was used as an intervention program in the present study.

Admitting the rising burden and negative consequences of LBP among nurses in our study population, an interactive LBP module was developed based on the Back School Program to suit the local context and provide local data for LBP prevention strategies. Specifically, we aimed to evaluate the effectiveness of this module among nurses with LBP in government hospitals in Penang, Malaysia by comparing disability scores between control and intervention groups. We hypothesized that an intervention module based on the Back School Program involving a combination of muscle strengthening, stretching, muscle mobilizing, and core stability exercises using gym balls would be effective in reducing symptoms of LBP among the nurses in our study population.

## 2. Materials and Methods 

### 2.1. Design

In a previous cross-sectional study, we identified that 898 out of 1292 (76.5%) nurses in the public hospitals of Penang suffered from LBP in 2016 [14]. As a continuation of this previous study, the present cluster-randomized experimental study was conducted over the course of three months from 1st September 2016 to 30th November 2016. The method described below was implemented throughout the entire duration of the study in accordance with the initial plan, having undergone no changes after the trial commenced.

### 2.2. Setting 

This intervention study was conducted in four out of six government hospitals in Penang, Malaysia. The state of Penang is located on the northwest coast of Peninsular Malaysia, allocated by Penang Island and Seberang Perai, and covers a 586 km^2^ area with a population of 1,902,116 as of 2016. The intervention program was held at Hospital Pulau Pinang and Hospital Balik Pulau (Penang Island), while the control group program was held at Hospital Seberang Jaya and Hospital Kepala Batas (Seberang Perai).

### 2.3. Participants

A total of 284 nurses with significant LBP were randomly selected from the selected government hospitals in Penang. Significant LBP was defined as persistent, disabling chronic LBP occurring for three consecutive months and self-rated as pain with an intensity of >20 mm on the visual analogue scale for pain [15]. Nurses meeting the following criteria were excluded from the study: those with cognitive disorders, those who were already under specialist care for LBP, those who had undergone previous surgeries within the last three months, those who were pregnant, and those with LBP due to traumatic conditions (e.g., LBP following an accident). 

Participants were given the option to withdraw at any point in time. The withdrawal criteria included pregnancy during the intervention period, injury during the intervention program, worsening back pain, changes in place of work during the intervention, and withdrawal due to personal option.

Ethical approval was obtained from the Human Research Ethics Committee of University Sains Malaysia (USM/JEPeM/15090308) and the Malaysian National Medical Research Register (NMRR-15-1668-27637). Permission to use the BACKS Tool was obtained from the Center for Collaborative Innovation of University Kebangsaan in Malaysia. A grant for this research was received from the Division of Research and Innovation of Universiti Sains in Malaysia. At each participating hospital, on-site permission for the nurses to participate in this research was obtained from the hospital director, the matron-in-charge, and the ward/clinic’s sister-in-charge. Verbal and written informed consent were obtained from participants preceding the intervention.

### 2.4. Sample Size Determination

The sample size was calculated using the two dependent means option in STATA SE 11 software (StataCorp LLC, College Station, Texas, TX, USA) with Oswestry Disability Scores (ODSs) as the outcome variable of interest. The correlation between the baseline and follow-up measurements was taken as 0.5 to determine the effectiveness of the intervention (range: 0.5–0.7). To achieve a power of 80% with 5% Type I error, a sample size of 142 per arm was calculated.

### 2.5. Randomization and Sampling

**Figure 1:**
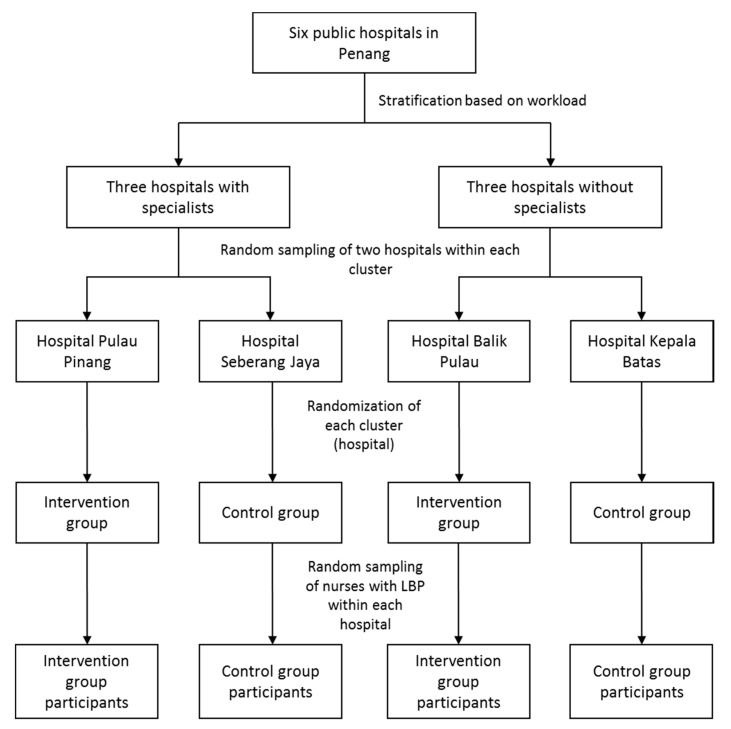
Randomization and recruitment of study participants.

The government hospitals were stratified into specialist and non-specialist hospitals to ensure that both groups were balanced in terms of work burden. Then, two hospitals were randomly selected from each cluster. A simple random sampling method was used to choose two of three hospitals (i.e., two with specialist services and two without specialist services) to ensure the representativeness of the sample and avoid any selection bias. Simple randomization was implemented to allocate a cluster of two out of three hospitals with specialist services and two out of three hospitals without specialist services into the intervention and control groups. One hospital from each cluster was assigned to the intervention group, and the other hospital from each cluster was assigned to the control group. Individuals who were unaffiliated with the present research drew lots for this process of allocation to the intervention and control groups. To avoid contamination in the intervention program, the selected hospitals were assigned to the intervention and control groups by different hospitals. Nurses from each selected hospital who were identified in our previous study as having LBP were randomly selected using a simple random sampling method. A schematic diagram of this selection process is depicted in Figure 1.

### 2.6. Intervention

In this cluster-randomized trial, an LBP module was developed based on the Back School Program, consisting of a health education module with an exercise method to overcome LBP and prevent LBP from worsening. This module was developed by a group of specialists, including a rehabilitation specialist, an occupational specialist, a public health specialist, and a physiotherapist. The main objective of this module is to help nurses increase their awareness of back pain care, improve their LBP, and subsequently promote a healthy lifestyle in the nursing workplace. This intervention module was designed to be a simple, sustainable, and affordable program that can be practiced at home without supervision.

The health education session consists of four sections: Section 1 consists of education on the structure and function of the backbone, Section 2 consists of education on LBP and work-related back pain, Section 3 consists of education on methods of preventing LBP among nurses, and Section 4 consists of an exercise method to address LBP among nurses. A one-day course was held at the auditorium of Hospital Pulau Pinang. During this course, each section of the module was presented by a rehabilitation specialist, an occupational specialist, a physiotherapist, facilitators, and researchers and discussed in detail among the respondents.

The exercise intervention package in this study was based on the Back School Program with the addition of a core stability exercise requiring the use of a gym ball. The Back School Program is a set of exercises aimed at improving mobility, flexibility, and strength. With the addition of a gym ball exercise, the intervention package developed for this study consisted of exercises for stretching, strengthening, mobilizing, and exercising core stability. The participants in the intervention group attended an exercise program for three sessions every alternate week over the course of the first six weeks. The duration of exercise per session was two hours. Exercise sessions were held from Monday to Thursday from 2:30 p.m. to 4:30 p.m. These sessions were held in the evening to allow ample time for nurses working morning shifts to attend the sessions after finishing work at their wards. The nurses were allowed to choose either one of the sessions each week so that the intervention did not disturb their ward duties. During the exercise sessions, the participants were instructed not to go beyond a comfortable range of movement.

The exercise sessions were held at the physiotherapy department of each hospital and conducted by a group of experienced physiotherapists in the presence of the researchers. In each session, three physiotherapists were assigned by the Department of Physiotherapy at Penang General Hospital to train the nurses and ensure that their exercise techniques were correct. In each session, the physiotherapists reviewed the nurses’ exercises and home diaries and facilitated their problem in doing exercise. 

A pamphlet with information regarding the exercise regime was given to all participants in the intervention group to help them carry out the exercise techniques in their homes. This pamphlet described the steps involved in every exercise conducted in this study.

### 2.7. Exercise Program 

**Figure 2:**
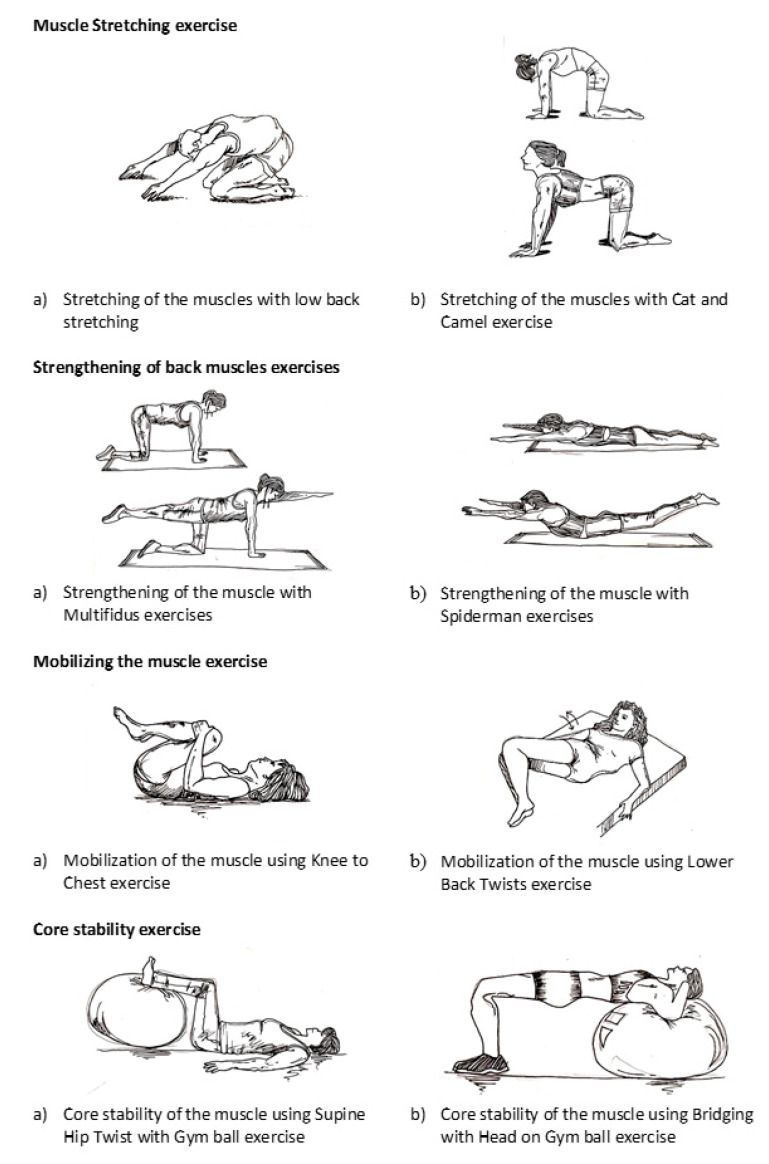
Exercise program.

The exercise program included muscle stretching, strengthening, mobilizing, and core stability exercises using a gym ball (Figure 2). The muscle stretching exercises included (a) low back stretching and (b) a cat and camel exercise. The low back stretching exercise required participants to sit on their knees and ankles, place their hands on their thighs, straighten their hands down their thighs to the floor in front of them, pull down and breathe out while maintaining this position for 30 to 60 s, and finally ride back up. This routine was to be repeated in two to four repetitions. For the cat and camel exercise, the participants were required to bear their bodies with both arms straight and with their knees in a bending position, stretch their backs by pushing them up, maintain this position for 15 to 30 s, return to the starting position, and finally press their abdomens toward the floor and lift their buttocks for 15 to 30 s. The routine was to be repeated in two to four repetitions.

The back strengthening exercises included (a) a multifidus exercise and (b) the Spiderman exercise. The multifidus exercise required the participants to bear their bodies with both hands and knees bent, keeping their hands under their shoulders and their knees under their hips. The participants were also instructed to make sure that their spines were in a neutral position, to keep their heads in line with their spines, and to breathe in and out. During exhalation, one leg was to be straightened and the opposite arm was to be in line with the spine. This position was held for 5 to 10 s and then repeated on the opposite side. The Spiderman exercise required the participants to lie down on the floor with their faces down and their stomachs flat on the floor. Next, both their arms and their legs were to be raised simultaneously and their chests were to be lifted so that their backs were off the floor. This position was maintained for 2 s before slowly returning to the starting position. This routine was repeated in 10 to 15 repetitions.

The muscle-mobilizing exercises included (a) a knee-to-chest exercise and (b) a lower back twist. In the knee-to-chest exercise, the participants were instructed to lie down and bend their knees up, pulling both knees slowly back toward their chests; this movement was repeated ten times. In the lower back twist exercise, they were instructed to lie on their backs, bend and press their knees, and move their knees from side to side ten times.

Finally, the core muscle stability exercises included: (a) a supine hip twist with a gym ball and (b) a bridge exercise with the head on a gym ball. For the supine hip twist, the participants were instructed to lie on the floor with their hips and knees bent 90 degrees and their feet set upon the gym ball. Next, they were instructed to tighten and hold their abdominal muscles. Then, they were to lower their knees to one side of the hips in a slow, controlled manner, avoiding contact with the floor. Finally, they were to raise their knees back to the starting position and repeat the exercise on the opposite side. These exercises were repeated 10 to 20 times on each side. The bridge exercise required the participants to align their shoulders at the top and middle of a gym ball with both their hands at their ears. Then, they were to place both feet on the floor and keep their thighs parallel to their bodies, maintaining a straight line from neck to knee. The participants were instructed to hold this position for 3 to 5 s and repeat the exercise 10 to 20 times.

### 2.8. Control Group

The control group was given standard care and advice for a sedentary lifestyle by an attending doctor without referring to any specific module. Then, the controls were given the same questionnaire (the Oswestry Disability Questionnaire) and assessed simultaneously with those in the intervention group. A researcher asked the participants if any problems arose to improve the relationship between the researchers and the respondents. The respondents were advised to feel free to contact the researcher at any time if they needed assistance throughout the intervention program.

### 2.9. Research Instrument and Assessment

The Oswestry Disability Questionnaire is a component of the BACKS Tool. It is a self-completed questionnaire containing ten subheadings intended to examine pain intensity, ability to lift, ability to care for oneself, ability to walk, ability to sit, sexual function, ability to stand, social life, sleep quality, and ability to travel [16]. Six statements follow each subheading, describing various risk scenarios relating to the matter. Each of the subheadings is scored on a scale of 0–5, with the first statement scoring 0 (indicating the least risk of disability) and the last statement scoring 5 (indicating the most severe disability) [16]. An index (ranging from 0 to 100) is obtained by summing up the scores for all the answered questions and then multiplying this sum by two. Zero is indicative of no disability, and 100 is indicative of the maximum degree of disability possible. This questionnaire was translated to the Malay Version and validated by Zhueng [17]. The internal consistency of this questionnaire was excellent, with a Cronbach’s alpha of 0.938. 

The study protocol started with a baseline assessment, which was followed by an intervention package of health education sessions and exercises for back pain, some of which involved a gym ball. The respondents attended the intervention program for six weeks. Diary reviews and follow-up assessments were conducted at the ends of the third and sixth weeks post intervention program. Adequate time was allowed for participants to respond to the questionnaire at each assessment session.

Baseline assessments of the intervention and control groups were conducted on separate consecutive days. All participants completed the Malay version of the Oswestry Disability Questionnaire, which was administered by a trained interviewer to small groups of eight to ten respondents. This questionnaire takes approximately 15–20 min to complete. At the ends of the third and sixth weeks of the intervention, the participants were required to complete the questionnaire again. The assessments were completed on separate days for the intervention and control groups. The participants’ home diaries and any problems or adverse events reported in these diaries were evaluated on the assessment days.

### 2.10. Monitoring Compliance

The exercise programs were conducted in four sessions per week, and each nurse was required to attend any two of these sessions per week according to their convenience. The participants were provided with reassurance and motivation to comply with the exercises during each session. Physiotherapists demonstrated, observed, and corrected the performance of the exercises. Clear instructions concerning how to perform the exercises were provided in a manual and a CD that were distributed to each participant in the intervention group. Participants were required to keep logs of their performance of the exercises in a book that was provided to them, and these logs were assessed by the physiotherapist during their weekly appointments. The participants were also required to perform the exercises in front of the physiotherapist at least once per week. This allowed the physiotherapist to confirm the proper execution of these exercises and correct any improper techniques. These regular reviews also served to reinforce the importance of exercising and encourage consistency. For the nurses who could not attend these sessions at the stipulated times, new schedule arrangements were made at follow-up.

### 2.11. Method of Statistical Analysis

In this cluster-randomized trial, the units of analysis were the individual nurses rather than clusters. Data entry and statistical analysis were conducted in IBM SPSS version 22 (IBM Corp, Armonk, New York, NY, United States). The collected data were checked, explored, and cleaned prior to analysis. Each participant’s age was categorized as less than 30 years old, 30 to 40 years old, or more than 40 years old. Other variables included body mass index (underweight, normal weight, overweight, or obese), gender (male or female), marital status (single or married), education level (having a diploma or education above a diploma), and number of children (none, one to three, and four or more). All variables were presented as frequencies (*n*) and percentages (%). Sociodemographic characteristics were compared between groups using a chi-square test.

The participants’ ODSs were measured as a numerical variable. The resulting distribution was examined using a histogram overlaid with a normal curve and tests of normality (the Kolmogorov–Smirnov test and the Shapiro–Wilks test). Descriptive statistics concerning the participants’ ODSs were presented as means (SDs), and baseline scores were compared between the control and intervention groups using an independent samples t-test. 

A repeated measure analysis of variance was conducted to determine the effectiveness of the intervention module by assessing two effects: a time-intervention effect and a time effect. Normality of residuals, homogeneity of variance, and assumptions of compound symmetry were examined as well. For all the aforementioned statistical tests, a *p*-value of less than 0.05 was set as the level of significance.

## 3. Results

**Figure 3:**
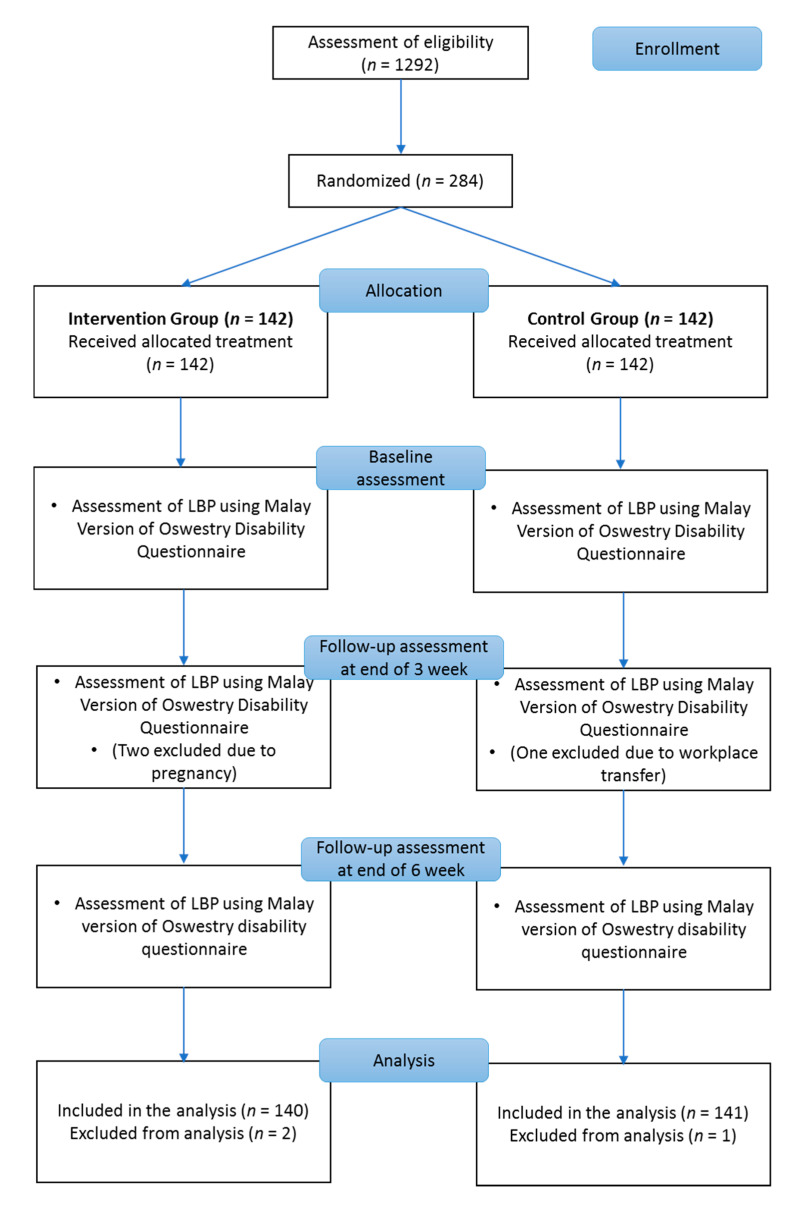
Consolidated Standards of Reporting Trials (CONSORT) flowchart of study participants.

Out of 284 nurses (intervention group: 142 nurses, control group: 142 nurses), only 281 participants (intervention: 140 nurses, control: 141 nurses) completed the study. Two nurses in the intervention group discontinued the intervention due to pregnancy, and one nurse in the control group was unable to continue due to a transfer to another state (Figure 3). No adverse events were reported by any of the participants throughout the entire intervention period.

Most of the nurses were aged less than 30 years old in both the intervention group (72, 50.7%) and the control group (65, 45.8%). Further, the body mass index of most participants in both groups were within the normal (intervention group: 40.8%, control group: 52.1%) or overweight range. The majority of the nurses were female and married with no children. The sociodemographic characteristics of the participants are described in Table 1.

### 3.1. Effectiveness of the Interactive LBP Module

#### 3.1.1. Between-Group Difference Over Time (Time-Intervention Interaction)

A significant difference in mean ODSs was observed between the control and intervention groups over time. At the end of the third week, the participants in the intervention group showed a significantly lower ODS than the control group (Cohen’s *d* = 0.33). A similar finding was observed at the end of the sixth week of the intervention (Cohen’s *d* = 1.02; Table 2).

#### 3.1.2. Within-Group Changes Over Time (Time Effect)

The multivariate test showed that the F-statistics probability was significant, indicating significant within-group changes over time. A pairwise comparison showed that the mean ODS of the intervention group significantly improved from baseline to the third week (Cohen’s *d* = 1.27), from baseline to the sixth week (Cohen’s *d* = 1.40), and from the third week to the sixth week of the intervention (Cohen’s *d* = 0.72).

There was no significant reduction in the mean ODS of the control group from baseline to the third week (Cohen’s *d* = 0.11) or from the third week to the sixth week of the intervention (Cohen’s *d* = 0.18). However, the mean ODS of the control group did significantly reduce from baseline to the sixth week of the intervention (Cohen’s *d* = 0.09; Table 3).

## 4. Discussion

Nurses play a major role in patient management, particularly for warded patients. The scopes of their jobs expose them to a high risk of developing back pain. Various factors, such as sociodemographic, work-related, and lifestyle factors, have been reported to be associated with LBP [18,19].

Both groups in the present study demonstrated good compliance. None of the participants within the intervention group withdrew from the study; this reflects the acceptability of the prescribed exercise regime. A comparison of ODSs at the third and sixth weeks of the intervention revealed significantly lower scores within the intervention group. The ODSs of the intervention group were found to change from the third week of the intervention until the end of the study. This reduction in scores indicates that the intervention package had a positive impact in terms of disability reduction among nurses with LBP in Penang public hospitals.

The most effective treatments for LBP involve a multidisciplinary approach. The most popular of these techniques is the Back School Program, which is an educational program involving physical exercises. In a study by Sahin et al. [20], symptoms of back pain and functional disability were significantly reduced after an exercise and educational program using the Back School method. This study differed from the present study in terms of the intervention method, in that the Back School module used in the previous study included a combination of physical therapy and a post-assessment taking place three months post-intervention. The Back School module used in the present study focused on exercise, including the use of a gym ball. This module was effective in reducing LBP, as evidenced by a reduction in the participants’ ODSs post-intervention.

Exercise therapy has been demonstrated to have a positive effect [21], even when there are no specific standards regarding the types of exercise that must be used. Exercise coupled with additional bodyweight resistance training has been suggested to be most appropriate to reduce the pain [22]. Static core muscle strengthening and spinal stabilization have also been recommended for the treatment of LBP [23,24]. In a recent systematic review and meta-analysis examining the effectiveness of exercise interventions for the treatment of chronic LBP, it was found that exercise has significant benefits in the treatment of nonspecific chronic LBP. Although the magnitude of this effect was small, exercise was more effective than conservative therapies [25]. 

A study by Chung et al. [26] indicated that stabilization exercise involving a gym ball is associated with a significant decrease in functional disorder among patients with chronic LBP. Compared to general stabilization exercises, stabilization exercises using gym balls were followed by greater improvements in functional disorder indexes. This study was similar to the present study, in that a gym ball was used in the muscle stabilization exercises. Compared to floor exercises, exercises using gym balls have been found to be superior in terms of increasing the activity of all trunk muscles [27]. Hides et al. [28] suggested that the spinal erector muscles play essential roles in trunk stability. Strengthening of the extensor muscles is important for patients with chronic LBP because weakened lumbar extensor muscles could lead to weakened lumbar flexor muscles [29]. Dynamic lumbar stabilization exercises using a gym ball cause pain reduction through improvements in trunk muscle strength, endurance, balance, and flexibility [30]. Simple and sustainable exercises such as these could be practiced in back pain reduction programs and even at home. 

Other exercise modalities have been studied previously and shown to have similar effectiveness in terms of reducing pain and disability among participants with LBP. Cruz-Díaz et al. [31] examined the effectiveness of practicing pilates in patients with chronic nonspecific LBP and showed that 12 weeks of pilates caused significant improvements in disability and pain. Further, a review by Eliks et al. [32] reported that pilates-based exercises yield beneficial results for patients with chronic LBP and can therefore be utilized as a therapeutic option.

## 5. Limitations

Interventions for LBP among nurses should involve various departments and areas of expertise, including government agencies and hospital departments such as orthopedic departments, medical departments, rehabilitation and physiotherapy units, and pain management units. The present study only involved rehabilitation therapists and physiotherapists. Therefore, all the collective bodies of knowledge represented in the literature on LBP management should be integrated to inform practice and integrate information that is missing from the present study. The integration of modalities used in diverse areas of expertise will ensure good outcomes in LBP treatment. 

Although the nurses participating in this study were grouped based on the geographical locations of their hospitals, the risk of data contamination was not guaranteed. The social gathering of nurses through events, workplaces, phone calls, and social media networks was a potential threat to the validity of this study, as it may have resulted in data contamination. This factor was beyond our control. Further, only nurses from public hospitals in Penang, Malaysia were included in this study. Therefore, generalizations to other groups of nurses should be made with caution. 

The Oswestry Low Back Pain Disability Questionnaire is considered the gold standard for the assessment of the functional outcomes of LBP patients. Nevertheless, this assessment is subjective, and the lack of objective assessments of muscle strength, pain (e.g., analogue pain scales), range of mobility, or any other objective variables served as a limitation in this study. 

## 6. Conclusions

Based on the discussion above, we can conclude that the combination of muscle stretching, muscle strengthening, muscle mobilization, and core stability exercises using a gym ball has proven effective for the treatment of LBP. The interactive LBP module developed in this study was effective in improving overall LBP among nurses at Penang Public Hospital. This intervention module was constructed to ensure that nurses could complete their exercises using simple, suitable, and sustainable techniques in a home setting with limited facilities. The introduction of interactive LBP modules could impact the physiotherapeutic management of back pain by encouraging new initiatives that may ultimately improve quality of care.

## Figures and Tables

**Table 1:** Sociodemographic characteristics of the participants in the intervention and control groups.

Variable		Intervention Group (*n* = 142)	Control Group (*n* = 142)	*p*-Value ^a^
Age (years)	Less than 30 30–40 More than 40	72 (50.7) 49 (34.5) 21 (14.7)	65 (45.8) 54 (38.0) 23 (16.2)	0.689
Body mass index	Underweight Normal Overweight Obese	20 (14.1) 58 (40.8) 50 (35.2) 14 (9.9)	8 (5.6) 74 (52.1) 46 (32.4) 14 (9.9)	0.064
Sex	Male Female	4 (2.8) 138 (97.2)	7 (4.9) 135 (95.1)	0.541
Marital status	Single Married	66 (46.5) 76 (53.5)	37 (26.1) 105 (73.9)	0.025
Education level	Diploma Above diploma	126 (88.7) 16 (11.3)	118 (83.0) 24 (16.9)	0.621
Number of children	0 1–3 More than 4	86 (60.6) 54 (38.0) 2 (1.4)	75 (52.8) 52 (36.6) 15 (10.6)	0.672

^a^ Chi-square test *p*-value.

**Table 2:** Comparison of mean Oswestry Disability Scores between the intervention (*n* = 140) and control groups (*n* = 141) with regard to time (time-intervention interaction effect).

Variable	Intervention Group	Control Group	Mean Difference (95% CI)	t-Stat (df)	*p*-Value
	Mean (SD)	Mean (SD)			
Baseline	34.62 (7.58)	32.47 (11.55)	2.14 (−0.16, 4.46)	1.83 (282)	0.068
Third week	28.04 (8.83)	31.46 (11.67)	−3.43 (−5.86, −0.99)	−2.77 (279)	0.006
Sixth week	20.98 (5.96)	30.55 (11.83)	−9.58 (−11.78, −7.34)	−8.56 (279)	<0.001

A between-group repeated measures analysis of variance was applied with regard to time. Assumptions of normality, homogeneity of variance, and compound symmetry were checked and fulfilled. Pillai’s Trace F (df) = 125.83 (2, 278), *p*-value < 0.001, partial η^2^ = 0.48.

**Table 3:** Comparison of Oswestry Disability Scores within each treatment group over time (time effect).

Comparison	Intervention Group (*n* = 140)		Control Group (*n* = 141)	
	Mean Difference (95% CI)	*p*-Value	Mean Difference (95% CI)	*p*-Value
Baseline—third week	6.51 (5.67, 7.36)	<0.001	1.06 (−0.55, 2.67)	0.343
Baseline—sixth week	13.57 (11.97, 15.17)	<0.001	1.97 (0.14, 3.80)	0.031
Third week—sixth week	7.06 (5.43, 8.68)	<0.001	0.91 (−0.72, 2.54)	0.538

A within-group repeated measures analysis of variance was applied. Pillai’s trace F (df) = 310.4 (2, 278), *p*-value = 0.001, partial η^2^ = 0.69.

## References

[1] Hoy D., March L., Woolf A., Blyth F., Brooks P., Smith E., Vos T., Barendregt J., Blore J., Murray C. (2014). The global burden of neck pain: Estimates from the global burden of disease 2010 study. Ann. Rheum. Dis..

[2] Aiken L.H., Clarke S.P., Sloane D.M., Sochalski J.A., Busse R., Clarke H., Giovannetti P., Hunt J., Rafferty A.M., Shamian J. (2001). Nurses’ reports on hospital care in five countries. Health Aff..

[3] Goetzel R.Z., Hawkins K., Ozminkowski R.J., Wang S. (2003). The health and productivity cost burden of the “top 10” physical and mental health conditions affecting six large U.S. employers in 1999. J. Occup. Environ. Med..

[4] Stewart W.F., Ricci J.A., Chee E., Morganstein D., Lipton R. (2003). Lost productive time and cost due to common pain conditions in the US workforce. Jama.

[5] Daraiseh N., Genaidy A.M., Karwowski W., Davis L.S., Stambough J., Huston R.I. (2003). Musculoskeletal outcomes in multiple body regions and work effects among nurses: The effects of stressful and stimulating working conditions. Ergonomics.

[6] Byrns G., Reeder G., Jin G., Pachis K. (2004). Risk factors for work-related low back pain in registered nurses, and potential obstacles in using mechanical lifting devices. J. Occup. Environ. Hyg..

[7] Wong T.S., Teo N., Kyaw M.O. (2010). Prevalence and Risk Factors Associated with Low Back Pain Among Health Care Providers in a District Hospital. Malays. Orthop. J..

[8] International Council of Nurses Positive Practice Environments: Quality Workplaces = Quality Patient Care. https://www.twna.org.tw/frontend/un16_commission/webPages_4/IND/1.pdf.

[9] Daynard D., Yassi A., Cooper J.E., Tate R., Norman R., Wells R. (2001). Biomechanical analysis of peak and cumulative spinal loads during simulated patient-handling activities: A substudy of a randomized controlled trial to prevent lift and transfer injury of health care workers. Appl. Ergon..

[10] Maher C.G. (2000). A systematic review of workplace interventions to prevent low back pain. Aust. J. Physiother..

[11] Hartvigsen J., Lauritzen S., Lings S., Lauritzen T. (2005). Intensive education combined with low tech ergonomic intervention does not prevent low back pain in nurses. Occup. Environ. Med..

[12] Forssell M.Z. (1980). The Swedish Back School. Physiotherapy.

[13] van Tulder M., Becker A., Bekkering T., Breen A., del Real M.T., Hutchinson A., Koes B., Laerum E., Malmivaara A. (2006). Chapter 3. European guidelines for the management of acute nonspecific low back pain in primary care. Eur. Spine J..

[14] Ibrahim M.I., Zubair I.U., Yaacob N.M., Ahmad M.I., Shafei M.N. (2019). Low Back Pain and Its Associated Factors among Nurses in Public Hospitals of Penang, Malaysia. Int. J. Environ. Res. Public Health.

[15] Jones G.T., Johnson R.E., Wiles N.J., Chaddock C., Potter R.G., Roberts C., Symmons D.P., Macfarlane G.J. (2006). Predicting persistent disabling low back pain in general practice: A prospective cohort study. Br. J. Gen. Pract..

[16] Fairbank J.C., Pynsent P.B. (2000). The Oswestry Disability Index. Spine.

[17] Zhueng T.J. (2014). BACKS Tool: Tool to discriminate work-related chronic back pain among employees. Unpublished Ph.D. Thesis.

[18] Mekonnen T.H. (2019). Work-related factors associated with low back pain among nurse professionals in east and west Wollega zones, Western Ethiopia, 2017: A cross-sectional study. Pain Ther..

[19] Yoshimoto T., Oka H., Ishikawa S., Kokaze A., Muranaga S., Matsudaira K. (2019). Factors associated with disabling low back pain among nursing personnel at a medical centre in Japan: A comparative cross-sectional survey. BMJ Open.

[20] Sahin N., Albayrak I., Durmus B., Ugurlu H. (2011). Effectiveness of back school for treatment of pain and functional disability in patients with chronic low back pain: A randomized controlled trial. J. Rehabil. Med..

[21] Rainville J., Hartigan C., Martinez E., Limke J., Jouve C., Finno M. (2004). Exercise as a treatment for chronic low back pain. Spine J..

[22] Urquhart D.M., Hodges P.W., Allen T.J., Story I.H. (2005). Abdominal muscle recruitment during a range of voluntary exercises. Man. Ther..

[23] Ebenbichler G.R., Oddsson L.I., Kollmitzer J., Erim Z. (2001). Sensory-motor control of the lower back: Implications for rehabilitation. Med. Sci. Sports Exerc..

[24] Liddle S.D., Baxter G.D., Gracey J.H. (2004). Exercise and chronic low back pain: What works?. Pain.

[25] Searle A., Spink M., Ho A., Chuter V. (2015). Exercise interventions for the treatment of chronic low back pain: A systematic review and meta-analysis of randomised controlled trials. Clin. Rehabil..

[26] Chung S., Lee J., Yoon J. (2013). Effects of stabilization exercise using a ball on mutifidus cross-sectional area in patients with chronic low back pain. J. Sports Sci. Med..

[27] Imai A., Kaneoka K., Okubo Y., Shiina I., Tatsumura M., Izumi S., Shiraki H. (2010). Trunk muscle activity during lumbar stabilization exercises on both a stable and unstable surface. J. Orthop. Sports Phys. Ther..

[28] Hides J.A., Jull G.A., Richardson C.A. (2001). Long-term effects of specific stabilizing exercises for first-episode low back pain. Spine.

[29] Mayer T.G., Smith S.S., Keeley J., Mooney V. (1985). Quantification of lumbar function. Part 2: Sagittal plane trunk strength in chronic low-back pain patients. Spine.

[30] Mori A. (2004). Electromyographic activity of selected trunk muscles during stabilization exercises using a gym ball. Electromyogr. Clin. Neurophysiol..

[31] Cruz-Díaz D., Romeu M., Velasco-González C., Martínez-Amat A., Hita-Contreras F. (2018). The effectiveness of 12 weeks of Pilates intervention on disability, pain and kinesiophobia in patients with chronic low back pain: A randomized controlled trial. Clin. Rehabil..

[32] Eliks M., Zgorzalewicz-Stachowiak M., Zeńczak-Praga K. (2019). Application of Pilates-based exercises in the treatment of chronic non-specific low back pain: State of the art. Postgrad. Med. J..

